# Minimal Important Difference (MID) of the Dermatology Life Quality Index (DLQI): Results from patients with chronic idiopathic urticaria

**DOI:** 10.1186/1477-7525-3-36

**Published:** 2005-05-20

**Authors:** Richard Shikiar, Gale Harding, Michael Leahy, Richard D Lennox

**Affiliations:** 1 MEDTAP International, Inc., Seattle, WA, USA; 2 MEDTAP International, Inc., Bethesda, MD, USA; 3 Aventis Pharmaceuticals, Inc., Bridgewater, NJ, USA; 4 Psychometric Technologies, Inc., Hillsborough, NC, USA

## Abstract

**Background:**

The Dermatology Quality Life Index (DLQI) has seen widespread use as a health-related quality of life measure for a variety of dermatological diseases. The purpose of this study was to estimate the minimal important difference (MID) on the DLQI for patients with chronic idiopathic urticaria (CIU).

**Methods:**

Data from 2 Phase III clinical trials of patients (N = 476 for Study A; N = 468 for Study B) with CIU were analyzed separately to estimate the MID for the DLQI for these populations. Both distributional based and anchor based approaches were used for deriving estimates. The anchor based approach relied upon patient self assessments of pruritus severity; the distributional based approaches relied upon estimating the standard error of measurement, as well as one-half the standard deviation of the DLQI from each study.

**Results:**

The distributional approaches resulted in estimates of MID ranging from 2.24 to 3.10 for the two studies. The anchor based approach resulted in estimates of 3.21 and 2.97 for the two studies.

**Conclusion:**

An MID for the DLQI in the range of 2.24 to 3.10 is recommended in interpreting results for patients with CIU.

## Background

Skin disease has long been recognized as having an adverse psychosocial impact on patients [1-3]. During the past decade, the formal assessment of patient health-related quality of life (HRQL) has been included in studies to assess the management of chronic skin disease and evaluate new treatments. The Dermatology Life Quality Index (DLQI), in particular, has been used in a number of studies of dermatological diseases including eczema, psoriasis, and chronic idiopathic urticaria (CIU) to evaluate the impact of treatment in these patient populations [4-9]. The DLQI was originally developed as a brief questionnaire for routine clinical use to assess the limitations related to the impact of skin disease and has been shown to be responsive to clinical changes in a study of dermatology [9].

While the DLQI and other outcomes measures provide a useful benchmark by which to evaluate the effectiveness of treatment, there has been a growing interest among clinicians and regulatory agencies, such as the U.S. Food and Drug Administration, in identifying meaningful change in HRQL. The concept of the minimal important difference (MID) refers to the smallest difference in a score that is considered to be worthwhile or important [10]. Juniper and colleagues [11-13] define a minimal clinical important difference as "the smallest difference in a score...which patients perceive as beneficial and which would mandate, in the absence of troublesome side effects and excessive cost, a change in the patient's management". For clinicians, it could be used as a threshold by which they recommend a therapy to their patients.

The purpose of this study is to estimate a MID for the DLQI in patients with CIU, a chronic skin condition defined by recurrent pruritic welts or wheals.

## Methods

Data for the analyses are based on two Phase III randomized, double-blind, parallel group, placebo-controlled multi-center clinical trials that were conducted to assess the efficacy and safety of fexofenadine HCl in the treatment of CIU. The two studies are labeled Studies A and B, and were similar in design. Details of these studies are reported elsewhere [5,6]. The primary objective of both studies was to assess the clinical efficacy and safety of a range of fexofenadine HCl doses (20, 60, 120, and 240 mg bid) compared to placebo for the relief of CIU symptoms. Both studies involved a 24-hour, single-blind placebo lead-in, followed by a four-week, double-blind treatment period. Each study included three site visits approximately two weeks apart. A secondary objective was to assess health-related quality of life (HRQL) among study subjects using the DLQI. The analyses described in this report do not evaluate treatment effects on HRQL. This report focuses on examining the MID of the DLQI among patients with CIU who participated in the two clinical trials.

### Subjects and inclusion criteria

Male and female subjects aged 12 to 65 years with a diagnosis of CIU, defined as the presence of urticarial wheals for at least 3 days per week for six consecutive weeks, were eligible to participate in these studies. In addition, subjects had to have a minimum of one to five wheals with moderate to severe itching during the previous 12 hours. A total of 476 and 468 subjects participated in Studies A and B respectively. Both studies recruited subjects from multiple clinical sites in the U.S. and Canada.

### Measures

#### Dermatology Life Quality Index (DLQI)

The DLQI was used to assess health-related quality of life among study participants. Subjects were asked to complete the DLQI at three scheduled study visits: baseline, week 2, and week 4 (end of study). The DLQI is designed to assess the impact of a wide range of skin disease on patient health-related quality of life (HRQL) [9]. It consists of ten items and covers six domains including symptoms and feelings, daily activities, leisure, work and school, personal relationships, and treatment. Response categories include "not at all," "a little," "a lot," and "very much," with corresponding scores of 0, 1, 2, and 3 respectively; the response "not relevant" (and unanswered items) are scored as "0". A total score is calculated by summing the score of all items, resulting in a maximum score of 30 and a minimum score of 0. Scale scores are calculated for each domain. Higher scores indicate poorer HRQL (i.e., more impairment).

#### Patient-assessed Pruritus severity

The primary efficacy measure for the clinical trial was the change in mean pruritus score over the four-week treatment period. Patients assessed urticarial symptom severity reflectively over the last 12 hours and recorded scores in a daily diary twice a day, in the morning and evening, just before taking their medication. Pruritus severity was rating on a scale of 0 to 4, where 0 = none; 1 = mild, not annoying or troublesome; 2 = moderate, annoying and troublesome, may interfere with sleep/daily activities; 3 = severe, very annoying, substantially interfering with sleep/daily activities; and 4 = very severe, warrants a physician visit. A daily score for patient-assessed pruritus severity was calculated as the average of the morning and evening assessments made each day. Change in mean pruritis severity was calculated as the difference between scores for baseline and end of study.

### Data analysis

We used two approaches to determine the MID for the total DLQL score including a distributional criterion approach and an anchor-based approach. Our primary approach was based upon distribution-based methods based on Wyrwich's work using the standard error of measurement (SEM) [14,15] and on the standard deviation of the measure of interest [16]. The SEM describes the error associated with the measure and is estimated by the standard deviation of the measure multiplied by the square root of one minus its reliability coefficient. The advantage of using the SEM is that it is not sample dependent due to the inclusion of the sample's reliability and variability in the SEM computational formula. Therefore in repeated samples drawn from the same population, the SEM values should be equivalent, except in cases where particular samples have a large number of subjects on the extreme ends of the distribution. Based on evidence supported by Wywrich, a one-SEM criterion was used to reflect a minimal clinically important difference in individual patient scores. Baseline assessments of total DLQI scores were used to calculate the SEM. More recently, there has been discussion [16] that a number of studies have demonstrated that one-half a standard deviation of a measure represents a good approximation of the minimally important difference, so this distributional approach was used as well.

As a means of confirming findings using the SEM-criterion approach, we also used an anchoring technique, whereby we examined changes in the DLQI total score by changes in disease severity using the patient-assessed mean pruritus score. Clinical meaningful change using this approach was defined as the difference in mean change of the DLQI total score for patients classified as "responders" and the mean change score for patients classified as "non-responders." Responders were defined as those with mean change score in pruritus severity greater than or equal to one, while non-responders were defined as those with a change score of less than 1 and equal to or greater than 0. Those with a mean change score in pruritus severity of less than zero (i.e., condition worsened) were not included in the analysis. Given that Study A and Study B were two independently conducted studies, results were analyzed separated.

## Results

### Distribution criteria

A total of 403 and 423 assessments were obtained for the DLQI total score at baseline for Studies A and B respectively. As can be seen in Table 1, mean total score for the DLQI at baseline was 9.64 (SD = 6.19) for Study A and 9.32 (SD = 5.61) for Study B. The coefficient alphas for the DLQI total scores in Studies A and B were 0.87 and 0.84 respectively. Based on these findings, results from both Studies A and B demonstrate that the SEM for the DLQI total score is 2.24. One-half the standard deviation for Studies A and B were 3.10 and 2.81, respectively.

**Table 1:** Distributional Characteristics of Total DLQI Score at Baseline for Studies A and B

**Item**	**N**	**Floor (%)**	**Ceiling (%)**	**Mean**	**SD**	**Cronbach's Alpha**	**SEM**
**Study A**							
Symptoms/Feelings	403	0.25	11.66	3.49	1.49	.	.
Daily Activities	403	24.07	3.72	1.92	1.69	.	.
Leisure	403	37.72	3.72	1.46	1.64	.	.
Work and School	403	27.05	15.14	1.21	1.01	.	.
Personal Relationships	403	53.85	2.73	1.04	1.53	.	.
Treatment	403	63.03	3.23	0.52	0.79	.	.
Total DLQI	403	2.98	0.25	9.64	6.19	0.87	2.24
							
**Study B**							

Symptoms/Feelings	423	0.24	9.69	3.48	1.39	.	.
Daily Activities	423	23.88	2.84	1.84	1.60	.	.
Leisure	423	37.35	4.02	1.38	1.56	.	.
Work and School	423	30.97	16.08	1.15	1.04	.	.
Personal Relationships	423	53.19	2.36	0.96	1.39	.	.
Treatment	423	62.65	2.84	0.51	0.76	.	.
Total DLQI at Baseline	423	0.24	0.47	9.32	5.61	0.84	2.24

Floor = percent who answered minimum value. Ceiling = percent who answered maximum value.

### Change over time to disease improvement

A total of 319 patients from Study A and 359 patients from Study B had both a baseline and end-of-study assessment for the DLQI total score. Of these, 9 patients from Study A and 7 patients from study B were excluded from the analysis, since their health worsened over the course of the study. Among the 310 patients from Study A included in the analysis, 150 (48%) were classified as "responders," while 160 (52%) were classified as "non-responders". Among the 352 patients from Study B included in the analysis, 208 (59%) and 144 (41%) were classified as "responders" and "non-responders," respectively. As can be seen in Table 2, in Study A, the mean change in DLQI total scores from baseline to end of study was -7.06 (SD = 5.95) for "responders" and -3.85 (SD = 5.30) for "non-responders". While these findings suggest an improvement in quality of life for both "responders" and "non-responders," the magnitude of change among "responders" is nearly twice that of "non-responders". Similarly, findings from Study B indicate that the mean change in DLQI total scores from baseline to end of study was -6.94 (SD = 5.32) for "responders" and -3.97 (SD = 5.79) for "non-responders". Thus, the difference in change scores of the DLQI total score between "responders" and "non-responders" was 3.21 and 2.97 for Studies A and B respectively.

**Table 2:** Mean change in DLQI scores (baseline to end of study) for responders and non-responders as assessed by patient-reported pruritus severity score

	**Baseline**		**End of Study**		Mean Change in DLQI*	
	
	Responder Mean (SD)	Non-Responder Mean (SD)	Responder Mean (SD)	Non-Responder Mean (SD)	Responder Mean (SD)	Non-Responder Mean (SD)
Study A (N = 310)	9.39 (5.56)	9.33 (6.35)	2.33 (3.14)	5.48 (5.53)	-7.06 (5.95)	-3.85 (5.30)
Study B (N = 352)	9.13 (5.38)	9.10 (5.72)	2.18 (2.89)	5.13 (4.99)	-6.94 (5.32)	-3.97 (5.79)

*End of study minus baseline

## Discussion

This study determined that a change in the DLQI total score in the range of approximately 2.2 to 3.2 could be considered clinically relevant in patients with chronic idiopathic urticaria (CIU). Our results are based on two different approaches for determining the minimal important difference (MID), including a distributional approach and a patient-based anchoring technique. These findings represent conclusions drawn from two separate Phase III clinical trials to assess the efficacy and safety of fexofenadine HCl in the treatment of CIU. To the best of our knowledge, this is the first study to determine a MID for the DLQI that could be used to differentiate patients treated for CIU who experience clinically meaningful change from those who do not in both therapeutic trials and routine care settings.

Using a one SEM distributional-based approach, we found that the MID for the DLQI total score in this patient population is 2.24. The SEM describes the error associated with the measure and Wyrwich has been able to show that this approach closely mirrors results using an approach based on patient global assessment of change [14,15]. For our study, we used a one SEM threshold. However, threshold values of 1.96 SEM [17] and 2.77 SEM [15,17], also have been suggested for defining clinically meaningful change. While 1.96 represents the value on a standard normal curve associated with a 95% confidence interval, the 2.77 value incorporates a multiplier of two to adjust for sampling error when using data from two samples (test and retest) versus one. Since our analysis is based on Cronbach's alpha coefficients obtained from two independent samples, as opposed to test-retest reliability coefficients from a single sample, we did not make this adjustment to the calculation.

Using the one-half standard deviation approach, we estimate the MID to be 2.81 to 3.10 (for studies B and A, respectively). These estimates provide estimates that are slightly larger than the estimates obtained from the SEM approach.

Finally, our finding of the MID threshold of 2.24 -3.10 is supported by similar results obtained when we used an anchoring technique based on change in patient assessment of their pruritus severity. Using this technique, the threshold for clinical change was 2.97 and 3.21 for the two clinical trials respectively. While anchor-based measures are typically based on perceived changes using a global question to address overall health (i.e., patients are asked to rate the degree to which their overall health improved or worsened over the course of follow-up), such a measure was not included in either clinical trial used for our study. Our results are based on the change in patient assessment of symptoms related to the degree of itching that patients reported at baseline and end-of-study and thus may not accurately reflect an overall assessment of health. The fact that the estimated MID in this study is less than the improvement shown by the "non-responder" group (improvement = 3.85 and 3.97 in the two studies, respectively – see Table 2) is anomalous. It may well reflect the fact that the two placebo groups in the two clinical trials upon which this study were based [5,6] showed significant improvement, although significantly less improvement than the active treatment groups.

It should be noted that direction of change may be important in defining clinically meaningful change. There are reports to suggest that a smaller amount of change may be required to be considered clinically important when a patient is improving compared to worsening [18-20]. Our analysis of the MID based on patient assessment of CIU symptom change did not include patients who worsened over the course o the study, since less that three percent of the study sample reported worsening symptoms.

The magnitude of estimates of the MID on the DLQI for CIU are similar to the estimates that can be derived from data recently presented using the DLQI to assess change in patients with moderate to severe psoriasis. Using the tables provided in the article by Shikiar and colleagues [21], one can derive SEM estimates of the DLQI of 2.44 and 2.42, one-half standard deviation estimates of 3.4 and 3.36, respectively, for the two studies reported on in their manuscript. In addition, they demonstrated that the difference in change scores over time between non-responders and partial-responders was 2.48 in one study and 4.34 in a second study. With the exception of the last value cited, these results are generally in line with estimates obtained in the present study using CIU patients. In addition, another estimate of the MID of the DLQI for use in a general dermatologic population was found to be 5.0 [22].

## Conclusion

In conclusion, we were able to identify the MID threshold that could be used to determine meaningful clinical change in patients treated for CIU when using the DLQI total score to assess their quality of life. This threshold, in the range of 2.24 to 3.10, could be used to interpret meaningful change in both clinical studies comparing alternative treatments and within the context of continuing care in a clinical practice setting. Further validation of these results are needed to explore the MID of the DLQI in other dermatology-related conditions and skin disease.

## Authors' contributions

RS and GH jointly developed the analytical approach and shared responsibility for interpreting the results and preparing the manuscript. ML reviewed results of the analyses, suggested additional analyses, and helped prepare the manuscript. RL reviewed the manuscript and provided helpful suggestions for its revision.
